# The Prognostic Role of HPV Status Based on the Analysis of Outcomes of Combined Treatment of Oral Cavity and Oropharyngeal Cancers

**DOI:** 10.3390/medicina62101998

**Published:** 2026-10-15

**Authors:** Katarzyna Miśkiewicz, Michal M. Masternak, Zuzanna Bożek, Jakub Łatka, Joanna Katarzyna Strzelczyk, Karolina Gołąbek, Dorota Hudy, Wojciech Majewski, Anita Stanjek-Cichoracka

**Affiliations:** 1 Department of Otorhinolaryngology and Oncological Laryngology, Faculty of Medical Sciences in Zabrze, Medical University of Silesia, 41-800 Zabrze, Poland; zuziabozek@gmail.com (Z.B.); jakublatka@protonamil.com (J.Ł.); 2 Burnett School of Biomedical Sciences, College of Medicine, University of Central Florida, Orlando, FL 32827, USA; michal.masternak@ucf.edu; 3 Department of Head and Neck Surgery, Poznan University of Medical Sciences, 61-701 Poznań, Poland; 4 Department of Medical and Molecular Biology, Faculty of Medical Sciences in Zabrze, Medical University of Silesia, 40-055 Katowice, Poland; asia.strzelczyk@gmail.com (J.K.S.); kgolabek@sum.edu.pl (K.G.); dorota.hudy@sum.edu.pl (D.H.); 5 Radiotherapy Department, Maria Skłodowska-Curie National Research Institute of Oncology (MSCNRIO), 44-102 Gliwice, Poland; wmajewski1@poczta.onet.pl; 6 Department of Community Pharmacy, Faculty of Pharmaceutical Sciences in Sosnowiec, Medical University of Silesia, Jedności 8b, 41-200 Sosnowiec, Poland; anita.stanjek@sum.edu.pl

**Keywords:** head and neck cancer, oropharyngeal squamous cell carcinoma cancer, HPV, 3-year OS rates, 3-year LRC rates

## Abstract

*Background and Objectives:* The aim of the paper is to assess the impact of HPV DNA on the survival and cure rates of patients with newly diagnosed head and neck cancers. *Materials and Methods:* The study group consisted of 51 patients with squamous cell carcinoma of the oral cavity and oropharynx. All patients underwent primary surgical treatment, extended by adjuvant treatment in 33 patients (65%). Cases with cT2 (n = 23; 45%) and cN0 (n = 25; 49%) were the most common. In total, 27 patients (53%) were HPV-dependent, including 14 (27%) patients with squamous cell carcinoma of the oral cavity and 13 (25%) with squamous cell carcinoma of the oropharynx. The Kaplan–Meier method was used to assess 3-year overall survival (OS) and 3-year locoregional control (LRC), depending on tumor location and HPV status. *Results:* The 3-year OS rates in the group of HPV-dependent and HPV-independent patients with oral cavity tumors were 80% and 82%, respectively; *p* = 0.965. In the group of HPV-dependent and HPV-independent patients with oropharyngeal cancer, the 3-year OS rates were 100% and 67.5%, respectively; *p* = 0.045. In patients with oropharyngeal cancer, the 3-year LRC rates for HPV-dependent and HPV-independent patients were 100% and 59%, respectively; *p* = 0.059. In the group of patients with oral cavity cancer, 3-year LRC rates for HPV-dependent and HPV-independent subjects were 55% and 52%, respectively; *p* = 0.771. *Conclusions:* HPV-dependent patients with oropharyngeal cancer had better 3-year OS and LRC rates compared to HPV-independent patients. However, in the group of patients with oral cavity cancer, HPV status did not show prognostic significance.

## 1. Introduction

Oral cavity and oropharyngeal cancers are the most prevalent types of head and neck cancer [1]. Collectively, they account for nearly 500,000 cases annually, with approximately 200,000 of these cases resulting in death, of which >75% are located in the oral cavity (GLOBOCAN 2020) [1]. The high mortality rate, reaching nearly 50%, is associated with the poor education and limited knowledge of patients and primary care physicians about head and neck cancers, the non-specific symptoms that prompt patients to seek specialist medical attention, and the late diagnosis, often at highly advanced disease stages [2,3,4]. Human papillomavirus (HPV) is associated with the development not only of head and neck cancer but also of cervical cancer in women [5]. It is worth noting that numerous studies have evaluated vaccination for the prevention of head and neck cancer, demonstrating a lower prevalence of oral infection post-vaccination [6]. A similar approach is observed in cervical cancer, where vaccination has become the primary line of prevention [7,8].

In recent decades, in addition to the well-known risk factors for head and neck cancers, such as tobacco smoking and alcohol abuse, increasing reports have indicated an association between these malignancies and exposure to the human papillomavirus (HPV), mainly genotypes 16 and 18, which particularly concerns oropharyngeal tumors [4]. Furthermore, HPV-dependent cancers increasingly affect younger individuals—the mean age of onset for HPV-dependent cancers is 54 years, and 58 years for HPV-independent cancers [4]. However, HPV-dependent cancers are associated with a more favorable prognosis, as reflected by LRC and OS rates, than HPV-independent cancers due to their greater sensitivity to chemotherapy and radiotherapy [9].

The 3-year OS rates for patients with oropharyngeal cancer treated with primary surgery range from 54.9% to 93.7%, regardless of HPV status [10,11,12,13,14,15,16]. In turn, LRC rates range from 73% to 98.6% [11,15,16,17]. Patients with HPV-dependent oropharyngeal cancer have a significantly better prognosis than those with HPV-independent cancer, and the 3-year OS rates in this group range from 90.9% to 100% vs. 65.7% to 84.3%, respectively [13,14]. Studies of patients with HPV-independent oropharyngeal cancers showed 3-year OS rates ranging from 75.6% to 85.5% [18,19,20].

In patients with oral cavity cancers, 3-year OS rates are significantly worse than in patients with oropharyngeal cancers, ranging from 53.9% to 82.5% [21,22,23,24,25], and LRC rates range from 53% to 87.5% [26,27,28,29,30]. The relationship between HPV infection and OS and/or LRC rates in patients with oral cavity cancer is not so evident. Most studies have not shown any impact of infection on the prognosis of these patients. The 3-year OS rates in patients with HPV-dependent oral cavity cancers were 56.2%, compared with 53.9% in patients with HPV-independent cancers [31], while the 5-year OS rates were 45.3–58.8% and 57.2–60.3%, respectively [32,33,34].

The aim of the study was to assess the impact of HPV DNA on 3-year OS and LRC rates in the entire study group of patients with newly diagnosed head and neck cancers, as well as in groups stratified by primary tumor location.

## 2. Materials and Methods

### 2.1. Study Population and Specimen Collection

The study included 51 patients with newly diagnosed squamous cell carcinomas of the oral cavity and oropharynx who were treated with surgery and adjuvant radiotherapy at the Department of Otorhinolaryngology and Oncological Laryngology of the Medical University of Silesia in Katowice and the Maria Skłodowska Curie National Research Institute of Oncology in Gliwice between 2016 and 2021.

All participants provided written informed consent before inclusion in the prospective study. The research protocol was approved by the Bioethics Committee (Institutional Review Board on Medical Ethics, Approval No. KNW/0022/KB1/49/16 and No. KNW/0022/KB1/49/II/16/17). For laboratory processing, fresh tissue specimens were flash-frozen in liquid nitrogen and subsequently stored at −80 °C. The entire analytical workflow, including DNA isolation, HPV detection, and genotype determination, was conducted at the Department of Medical and Molecular Biology, Faculty of Medical Sciences in Zabrze, Medical University of Silesia.

Patients with local and/or nodal cancer recurrence who had previously undergone radiotherapy or radiochemotherapy were excluded from the study. Locoregional recurrence was defined as the biopsy-proven return of the disease at the primary tumor site (local) and/or the regional lymph nodes (regional) after a documented period of complete remission. Persistent disease was defined as any disease that was not completely eradicated at the completion of the primary treatment protocol (determined by clinical and radiological evaluation at [e.g., 3 months/12 weeks] post-treatment) and was classified as persistent disease and counted as a locoregional event on day zero of the survival analysis. Distant metastases were tracked independently. In the LRC analysis, patients who developed distant recurrence without prior or simultaneous locoregional failure were censored at the date of distant recurrence. The mean age of the study participants was 63 years (range 27–87 years; 12 women and 39 men). Tissue samples were collected during surgery at the Department of Otorhinolaryngology and Oncological Laryngology of the Medical University of Silesia in Katowice and the Maria Skłodowska-Curie University of Warsaw. Intraoperative tumor biopsy samples were assessed for the presence of human papillomavirus (HPV) genetic material. The stage of cancer progression was determined based on postoperative histological results, according to the TNM classification (7th and 8th editions of the UICC) [35,36]. Patients diagnosed between 2016 and 2017 were originally classified using the 7th edition of UICC, whereas those diagnosed from 2018 to 2021 were staged using the 8th edition of UICC, which introduced distinct staging criteria for HPV-positive oropharyngeal squamous cell carcinomas.

### 2.2. p16 Immunohistochemistry

Formalin-fixed, paraffin-embedded (FFPE) tissue blocks containing an adequate volume of tumor tissue were selected by a pathologist for immunohistochemical evaluation. The selected blocks were cooled on ice or refrigerated at 4 °C, sectioned at a thickness of 4 μm, and mounted on positively charged adhesion slides. Freshly cut sections were allowed to dry at room temperature for several hours or overnight, and subsequently incubated in an oven at 60 °C for a minimum of 2 h. Automated immunohistochemical staining for p16 was performed using the CINtec p16 Histology assay on a BenchMark ULTRA platform (Roche Diagnostics), strictly adhering to the manufacturer’s validated protocol. Following automated processing, the slides were dehydrated, cleared, and coverslipped using a standard mounting medium.

The stained specimens were evaluated under a light microscope by a pathologist. High-risk HPV positivity was defined by the presence of a strong and diffuse nuclear and cytoplasmic p16 immunoreactivity (continuous, block-like staining pattern) within the tumor cells, whereas cases failing to meet these specific criteria were classified as p16-negative.

### 2.3. DNA Extraction and Quantification

Tissue samples were homogenized with Lysing Matrix A (MP Biomedicals, Irvine, CA, USA) in Lyse T buffer from the Tissue DNA Purification Kit (Eurx, Gdańsk, Poland) on the FastPrep^®^-24 instrument (MP Biomedicals, Santa Ana, CA, USA). DNA was extracted from the homogenates using the Tissue DNA Purification Kit (Eurx, Gdańsk, Poland) protocol. Isolated DNA was assessed spectrophotometrically using NanoPhotometer Pearl (Implen, München, Germany), and the samples were stored at −20 °C until further analysis.

### 2.4. Detection and Genotyping of HPV

HPV detection and genotyping were performed using the GenoFlow HPV (Human Papillomavirus) Array Test Kit (DiagCor Bioscience Inc., Hong Kong) according to the manufacturer’s instructions. Detailed methodological procedures have been described in the previous study [37]. The assay is based on PCR amplification followed by flow-through hybridization using biotin-labeled primers and genotype-specific probes and allows the detection of 33 HPV genotypes. The following genotypes were classified as high-risk: 16, 18, 31, 33, 35, 39, 45, 51, 52, 53, 56, 58, 59, 66, 68, 73, and 82, whereas genotypes 6, 11, 26, 40, 42, 43, 44, 54, 55, 57, 61, 70, 71, 72, 81, and 84 were classified as low-risk. All PCR reactions were performed using the Mastercycler Personal Thermal Cycler (Eppendorf, Hamburg, Germany). The hybridization step was performed using the FT-PRO Flow-through System (DiagCor Bioscience Inc., Kowloon, Hong Kong). The results were obtained using the CapturePRO image capture system (DiagCor Bioscience Inc., Kowloon, Hong Kong). Patients with any detected variant of HPV were assumed to be HPV-related, and those with a negative result were considered HPV-independent.

### 2.5. Radiotherapy

Adjuvant radiotherapy was delivered using IMRT technique and was systematically proposed in cases of microscopically involved margins (R1), positive lymph nodes, or advanced local disease (T3–T4). In cases where the surgical intervention consisted of local excision only, adjuvant radiotherapy was applied routinely. The typical treatment regimen consisted of a risk-adapted approach, prescribing 50 Gy in 2 Gy fractions to low-risk volumes (elective lymph node stations), with a dose escalation up to 60–66 Gy for intermediate- and high-risk volumes (tumor bed and involved nodal regions). For patients with advanced disease stages (≥T4 or N+ presenting with extracapsular extension [ECE]) who had no contraindications to systemic therapy, concurrent cisplatin-based chemotherapy was administered during the course of radiotherapy. Regarding the concurrent systemic treatment, chemotherapy was tailored individually based on the patient’s age, performance status, and organ function. The high-dose 3-weekly regimen (100 mg/m^2^) of cisplatin was the preferred standard, while the weekly regimen (30–40 mg/m^2^) or radiotherapy alone was reserved for elderly patients or those with renal impairment. High-Dose 3-weekly cisplatin: this regimen was implemented as the primary standard of care for younger patients with excellent performance status (ECOG 0–1) and adequate renal and hematological function. Low-Dose weekly cisplatin: this alternative schedule was selected for patients with borderline performance status or those requiring a more manageable toxicity profile to ensure treatment compliance.

### 2.6. Statistical Analysis

The Kaplan–Meier method was used to assess OS and LRC rates, and the Chi2 test was used to assess the relationship between HPV status and clinical factors. A *p*-value ≤ 0.05 was considered statistically significant.

## 3. Results

### 3.1. Patient Characteristics

In the study group of patients, histological examination showed 28 (55%) squamous cell carcinomas of the oral cavity and 23 (45%) squamous cell carcinomas of the oropharynx. Among patients with oral cavity cancer, the most common location was the tongue (N = 24; 86%), including 7 (25%) tumors involving the floor of the mouth. The most common location of oropharyngeal cancers was the palatine tonsil (N = 17; 74%), with the remaining tumors located on the soft palate (N = 6; 23%). Surgical removal of oral cavity and/or oropharyngeal tumors was performed predominantly using the classic transoral approach (N = 38; 74.5%), and less frequently via an extended surgical approach through midline mandibulotomy (N = 13; 25%). Thirty-three patients underwent adjuvant treatment, with the largest group consisting of patients receiving adjuvant radiotherapy (N = 24; 47%). The group of twenty-four patients after adjuvant radiotherapy consisted of thirteen patients with oropharynx carcinomas and eleven patients with oral cavity carcinomas (Table 1).

The largest group consisted of patients with cT2 stage (N = 23; 45%), clinically without nodal metastases (cN0; 49%), and patients with G2 grade (N = 24; 47%). HPV-positive oral cavity or oropharyngeal cancer was diagnosed in 27 (53%) patients. Within this group, molecular genotyping identified HPV genotype 16 (HPV-16) in 21 patients, HPV genotype 51 (HPV-51) in 5 patients, and HPV genotype 73 (HPV-73) in 1 patient (Table 2). All 21 cases with the HPV-16 genotype demonstrated strong and diffuse p16 protein expression on immunohistochemistry, whereas the remaining cases (HPV-51 and HPV-73) were p16-negative.

### 3.2. Association Between HPV Status and Clinical and Morphological Features

The study evaluated the association between HPV status and primary tumor location (oral cavity vs. oropharynx), tumor grade (G), clinical local stage (cT), and/or nodal stage (cN), and the need for adjuvant radio/radiochemotherapy. HPV status did not affect the local (Chi2 = 0.96; *p* = 0.810) or nodal stage of the disease (Chi2 = 2.16; *p* = 0.826), nor the tumor grade (Chi2 = 7.01; *p* = 0.069). In the group of HPV-independent vs. HPV-dependent patients, HPV-independent patients were offered adjuvant treatment significantly more often than HPV-dependent patients (Chi2 = 4.15; *p* = 0.041) (Table 3).

We also performed an additional analysis including factors characterizing the study group and treatment with respect to overall survival (OS). Due to the small number of patients, we did not further subdivide the group. As none of the factors were significant in the univariate analysis, we did not perform a multivariate analysis (Table 4).

Instead, we conducted Kaplan–Meier survival analyses with log-rank tests for factors characterizing the study group, treatment, and HPV status, separately for oral cavity cancer and oropharyngeal carcinomas (Table 5 and Table 6). Only HPV status in oropharyngeal carcinomas remained significant.

### 3.3. Overall Survival and Locoregional Control Rates

The 3-year overall survival (OS) rate in the study group was 83%, and the 3-year locoregional control (LRC) rate was 68%. No statistically significant difference was found in 3-year OS depending on the primary tumor location (OS for oral cavity and oropharynx: 80% and 85%, respectively; *p* = 0.339). With regard to 3-year LRC rates, patients with oropharyngeal cancer had a more favorable prognosis than those with oral cavity cancer (LRC for oropharynx and oral cavity: 87% and 53%, respectively; *p* = 0.014). The 3-year OS rates for HPV-dependent and HPV-independent patients were 90% and 74%, respectively; *p* = 0.27. The 3-year LRC rates for HPV-dependent and HPV-independent patients were 76% and 53%, respectively; *p* = 0.393.

**Figure 1:**
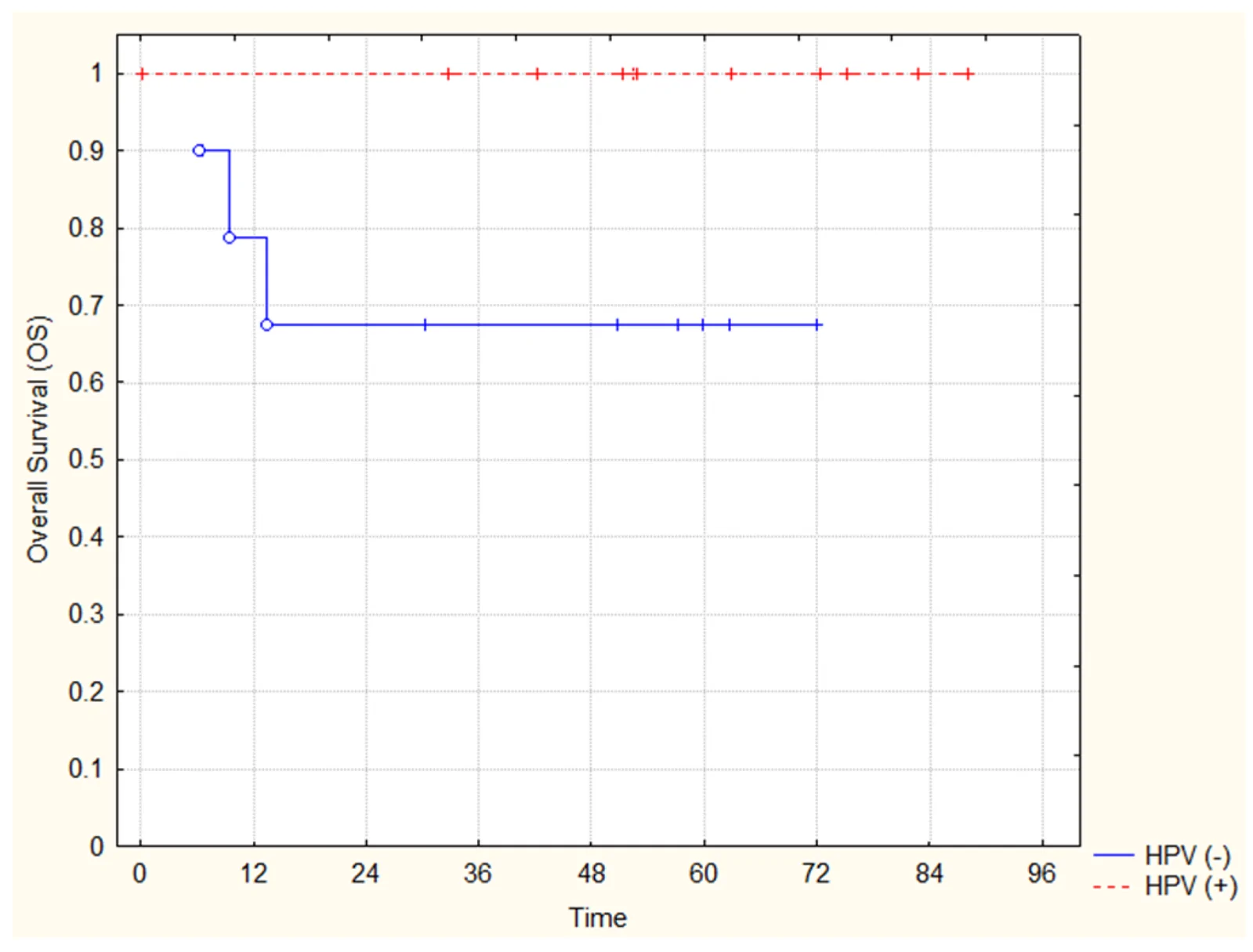
Three-year overall survival (OS) rates for patients with oropharyngeal cancer treated surgically, depending on HPV status.

**Figure 2:**
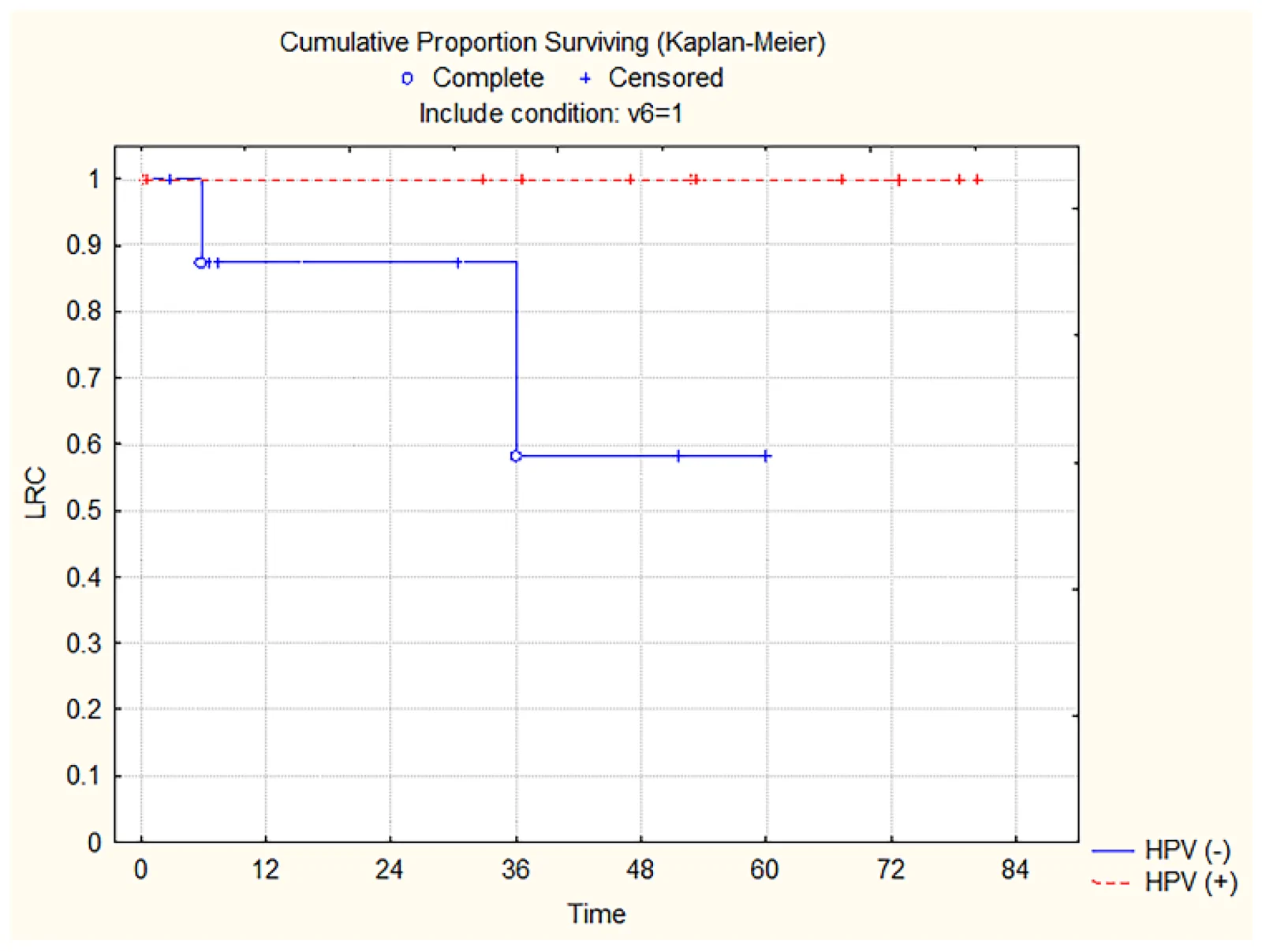
Three-year locoregional control (LRC) rates for patients with oropharyngeal cancer treated surgically, depending on HPV status.

The 3-year overall survival (OS) rate of patients with oropharyngeal cancer was 85%, and the 3-year locoregional control (LRC) rate was 87%. In the group of patients with HPV-dependent oropharyngeal cancer, better 3-year OS and LRC rates were observed compared to subjects with HPV-independent cancer (OS for HPV-dependent and HPV-independent: 100% and 67.5%, respectively; *p* = 0.045) (Figure 1); (LRC for HPV-dependent and HPV-independent was 100% and 59.5%, respectively; *p* = 0.059) (Figure 2).

**Figure 3:**
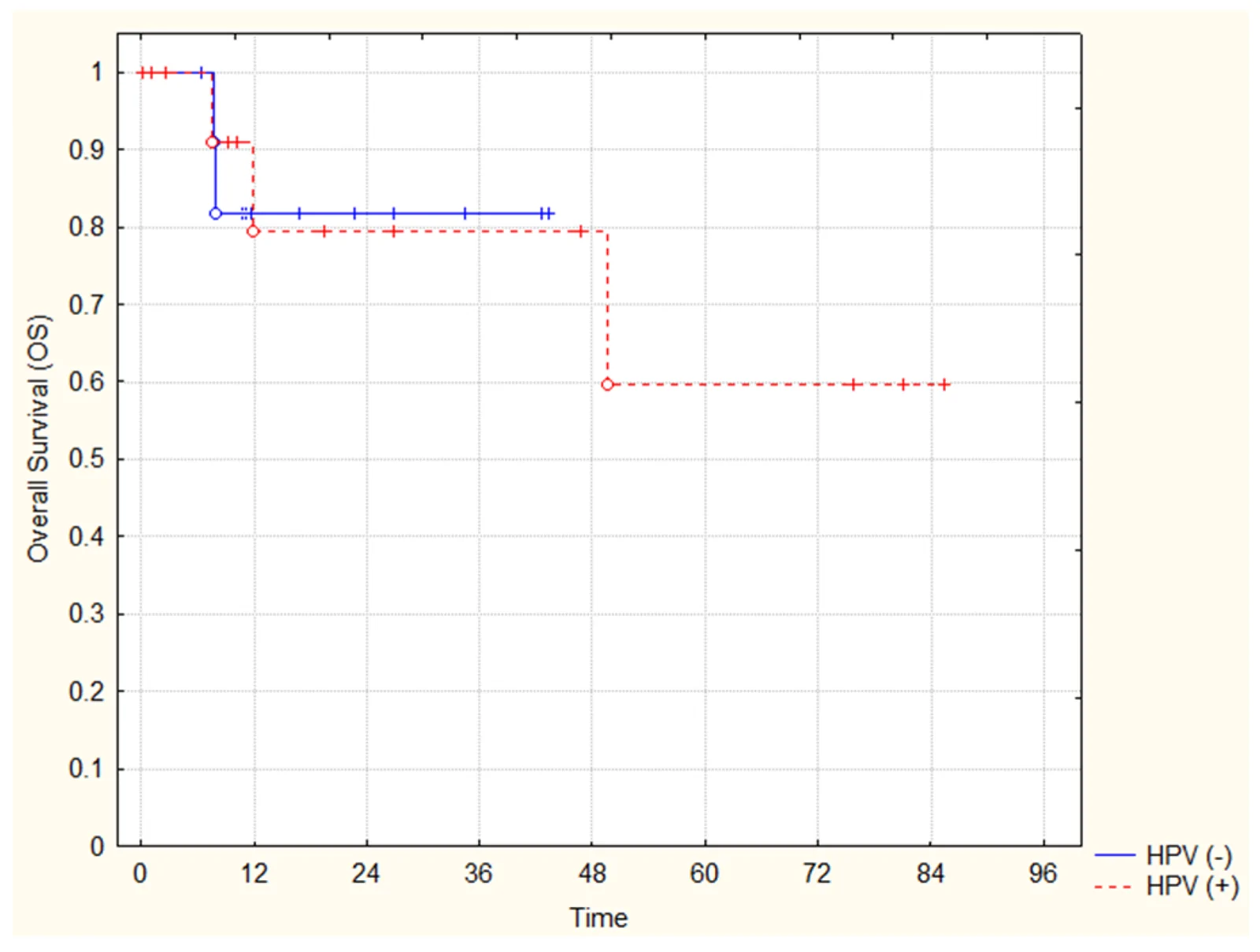
Three-year overall survival (OS) rates for patients with oral cavity cancer treated surgically, depending on HPV status.

**Figure 4:**
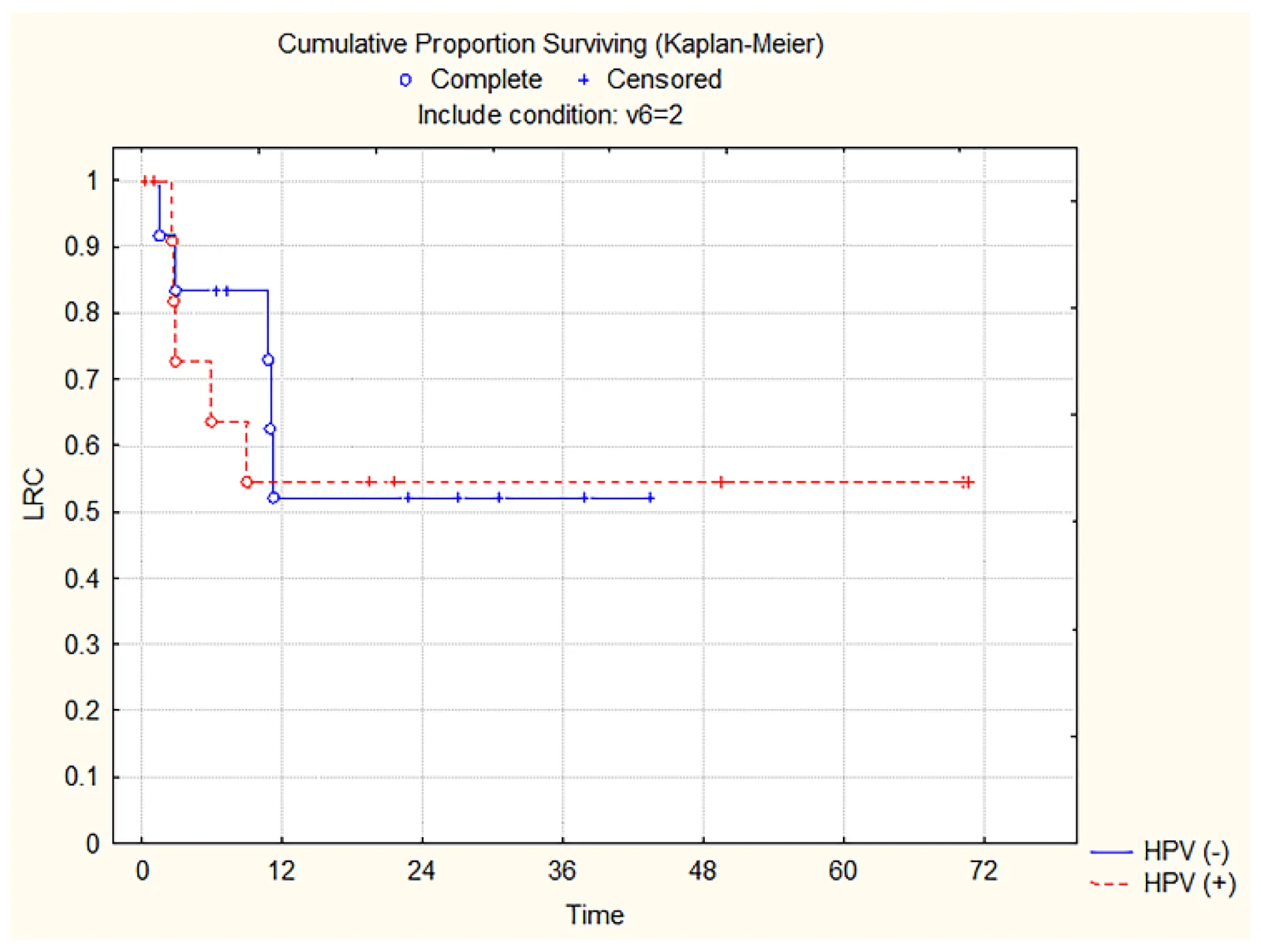
Three-year locoregional control (LRC) rates for patients with oral cavity cancer treated surgically, depending on HPV status.

The 3-year overall survival (OS) rate of patients with oral cavity cancer was 80%, and the 3-year locoregional control (LRC) rate was 53%. In the group of patients with HPV-dependent oral cavity cancer, no statistically significant improvement in 3-year OS or LRC rates was observed compared to patients with HPV-independent cancer (OS for HPV-dependent and HPV-independent was 80% and 82%, respectively; *p* = 0.965) (Figure 3); (LRC for HPV-dependent and HPV-independent was 55% and 52%, respectively; *p* = 0.771) (Figure 4).

To conclude, the study showed that HPV status had no prognostic significance for 3-year OS or LRC rates in the entire study group. At the same time, in the group of patients with oropharyngeal cancer, HPV status significantly affected their prognosis, improving it in HPV-dependent patients, as opposed to patients with oral cavity cancer in whom HPV status was not significant. The study also showed that HPV-independent patients were more often offered adjuvant treatment compared to HPV-dependent patients. The study showed that p16 expression status was not prognostic for 3-year OS in the entire study group.

## 4. Discussion

The aim of the paper was to assess the impact of HPV DNA as a prognostic factor on OS and LRC rates of patients with newly diagnosed head and neck cancers. The primary limitations of this study are the small sample size and the heterogeneity of the cohort regarding the use of adjuvant radiotherapy. These factors may have directly influenced the overall survival (OS) and locoregional control (LRC) rates, as well as the observation that HPV-independent patients more frequently received adjuvant treatment. Therefore, the results must be interpreted with caution. The 3-year OS of patients with oropharyngeal cancer reported in the literature ranged from 71% to 93.7% [12,13,14,16], regardless of HPV status or a surgical method, and the results of our study are consistent with this trend. Denittis et al. obtained the LRC rate of 73% in their retrospective study of 51 patients treated surgically who also received adjuvant radiotherapy [11]. In our study, most patients presented with T1 and T2 stages, while Denittis et al. assessed only patients with T3 and T4 stages, which may have influenced the difference as compared to our results. Between 1995 and 2007, Kim et al. examined a larger group of 133 patients, including those with oropharyngeal cancer (T1-T4). They obtained an LRC rate of 82.4% [15]. The results obtained by Patel et al. are particularly interesting. They analyzed a cohort of 80 patients with oropharyngeal cancer treated with laser microsurgery followed by adjuvant radiotherapy between 2000 and 2011. Although all study participants were in stage III or IV, they reported a 3-year LRC rate of 98.6% [16]. Moore et al. obtained a 97% LRC rate in a group of 66 patients with oropharyngeal cancer, mainly staged T1–T3, treated with surgery alone [17].

The relationship between HPV infection and oropharyngeal cancer has been well established in many publications for several years [9,38,39,40,41,42,43,44]. Furthermore, an increasing trend in the incidence of this cancer is evident [1,3,4,39]. In the United States alone, a 225% increase in cases of HPV-related oropharyngeal cancer was reported between 1988 and 2004. The proportion of HPV-related cancers also increased in patients with oropharyngeal cancer from 16.3% (1984–1989) to over 70% between 2000 and 2004 [40].

Other studies, including the research of Gillison et al., showed that in Europe and North America, HPV-dependent cancers accounted for 40.5% of oropharyngeal cancers before 2000, and 72.2% after 2005 [43]. Based on 3-year OS rates, we demonstrated a statistically better prognosis in patients with HPV-dependent oropharyngeal cancer compared to HPV-independent cancer (OS for HPV-dependent and HPV-independent was 100% and 67.5%, respectively). In the studies conducted by Saito et al., Hughes et al., and Kelly et al., 3-year OS rates in patients with HPV-dependent oropharyngeal cancer ranged from 90.4% to 94.4% [44,45,46]. In a similar study conducted by Jackson et al. on a cohort of 75 patients (>70 years of age), the 3-year OS was 81.5% [47]. It is lower than in the previously mentioned papers, likely due to the subjects’ advanced age. To compare, in studies on HPV-independent cancers, Yuan et al. and Kelly et al. obtained 3-year OS rates of 75.6–85.2% [18,19]. In the PREDICTOR-OPC study by Mehanna et al., involving 600 patients treated in the United Kingdom and Poland for oropharyngeal cancer initially with surgery followed by radiotherapy, an attempt was made to determine prognostic groups. The 3-year OS rate in patients with HPV-dependent oropharyngeal cancers ranged from 91.48% to 100% vs. 50% to 85.7% in HPV-independent patients. Their results are comparable to those obtained in our research [48].

For instance, Li et al. examined a group of 89 patients with T1 and T2 oropharyngeal cancer initially treated surgically using a surgical robot and obtained the LRC rate of HPV-dependent cancers of 96% compared to HPV-independent cancers (88.9%) [14]. Kim et al. and Wright et al. obtained a 2-year LRC rate of 93.3–97% in a group of 166 and 53 patients with HPV-dependent oropharyngeal cancer, respectively [49,50]. In a 3-year follow-up study by Shenker et al. involving 377 patients, including those treated with primary radiotherapy or radiochemotherapy, the LRC rate of 89.5% was achieved [51]. These results are consistent with our findings. In patients with HPV-independent oropharyngeal cancers, we achieved significantly worse outcomes in terms of the LRC rate (59.5%). In their study, Parhar et al., who observed only a group of patients with HPV-independent oropharyngeal cancer undergoing robotic surgery, obtained a 3-year LRC rate of 84.4% [20]. The above difference in results is probably due to the use of different surgical techniques by various authors. In patients with oral cavity cancer, the 3-year OS rate in our cohort was 80%, irrespective of HPV status. In a study by Fan et al. involving 92 patients, the 3-year OS rate was 82.5%, which was similar to our results [23]. Similarly, in a study by Shim et al., 86 patients with tongue cancer staged T1 and T2 treated surgically with optional adjuvant radiotherapy were examined, and survival rates of 80.8% were reported [25]. The higher survival rate is probably due to the fact that the study was limited to tongue cancers and T1 and T2 stages. A significantly lower 3-year OS rate than that observed in our study was found in a retrospective analysis by Spiotto et al. involving 4809 patients with locally advanced oral cancers (staged T3 and T4). The 3-year OS rate was 53.9% [21]. A similar OS rate (56.8%) was reported by Bera et al. in a 3-year follow-up of 287 patients [22]. Undoubtedly, the significantly worse survival was influenced by the inclusion of only patients with clinical stages 3 and 4. In their analysis, Bloebaum et al. reported the 5-year survival rate of only 41% [52]. The large discrepancy in the results, which were almost twice as poor as ours, may have been due to the longer follow-up (5 years), the larger number of patients and, above all, the significantly higher proportion of patients with stage 4 clinical disease (44% of the group) compared with our population. The small sample size had a significant impact on the obtained results, which must therefore be interpreted with great caution.

In our analysis, the LRC rate in patients with oral cavity cancer was 53%, regardless of HPV status. Hoffmann et al. achieved a 5-year LRC of 85% in a group of 75 patients with squamous cell carcinoma treated surgically with adjuvant radiotherapy [26]. In their group of 167 patients with the same tumor location, Mione et al. reported an 85.6% LRC rate in a 2-year follow-up, which is surprising given that 64% of their patients were in clinical stage IV. However, this finding may be attributable to the short follow-up period [27]. Daly et al. examined 37 patients with oral cavity cancer, of whom 30 received postoperative adjuvant radiotherapy and achieved a 67% LRC rate in a 3-year follow-up [28]. Collan et al. examined a group of 102 patients with squamous cell carcinoma of the oral cavity and/or oropharynx treated surgically with adjuvant radiotherapy and obtained a 5-year LRC of 92.2% [29].

While in the case of oropharyngeal cancer the association with HPV is relatively well established, this relationship is far less evident in oral cavity cancers. Similar to our findings, HPV infection did not have prognostic significance, as reported by Tangthongkum et al., who reported 3-year OS rates of 56.2% vs. 53.9% (HPV-dependent vs. HPV-independent) [31]. In 5-year follow-up studies by Wang et al., Doll et al., and Nauta et al., OS rates ranged from 45.3% to 58.8% vs. 57.2% to 60.3% (HPV-dependent vs. HPV-independent), and these differences were also statistically insignificant [32,33,34]. Compared with our results, the lower OS rates reported by the above authors may have been due to heterogeneity of patient groups, including the inclusion of patients treated with primary chemoradiotherapy, as well as a longer follow-up period.

Our study showed that expression of p16 status had no prognostic significance for 3-year OS rates in the entire study group. However, it should be remembered that the relatively small size of the study group may have influenced the data. In the PREDICTOR-OPC study by Mehanna et al., patients with oropharyngeal cancer who were p16+/HPV-dependent had a better 3-year OS rate than p16-/HPV-independent or p16+/HPV-independent patients [48]. Their results are comparable to those obtained by other authors [53,54,55,56].

To the best of our knowledge, there are no studies comparing LRC in patients with squamous cell carcinomas of the oral cavity, depending on HPV status. However, in their two papers, Lee et al. achieved 2-year local control (LC) of 80.7% vs. 95.1% (HPV-dependent vs. HPV-independent). At the 5-year follow-up, the rates were 84% vs. 86% (HPV-dependent vs. HPV-independent). In both papers, the neck control (NC) was calculated separately and was reported as 80.8% and 86.7% (HPV-dependent and HPV-independent, respectively) in the 2-year follow-up and 86% and 81% (HPV-dependent vs. HPV-independent, respectively) in the 5-year follow-up [38,39].

Our study showed that HPV-independent patients were offered adjuvant treatment more frequently after surgery compared to HPV-dependent patients. On the one hand, it is known that HPV-dependent patients have a lower clinical stage at diagnosis and have better OS and LRC rates than HPV-independent patients [57,58,59]. The lower clinical stage may have contributed to decisions not to administer adjuvant radiotherapy in the study group, which could explain the results. On the other hand, the HPV-independent patients could be offered adjuvant radiotherapy more often, even at lower stages, because of concerns about a worse prognosis. However, it should be remembered that the relatively small size of the study group may have influenced the data, and the decisions to omit adjuvant radiotherapy in HPV-dependent patients should be made with extreme caution, as it may contribute to an increased number of treatment failures in this cohort, as confirmed by other authors [58,60]. We are aware that our group of patients is small and heterogeneous in terms of radiotherapy. These facts may have influenced the results obtained, both in OS and LRC outcomes, as well as the finding that HPV-independent patients more frequently received adjuvant treatment.

We acknowledge the limitations of our study, particularly the small number of patients and the heterogeneous nature of the study group. However, such limitations are common in the literature, and our study is no exception. Our work serves as an important report on the successes and failures of oncological treatment in the most challenging and severe cases. We hope that, despite these limitations, our findings will contribute valuable insights into survival and locoregional control rates among patients with head and neck cancer, stratified by HPV status. It is possible that in the future, treatment outcomes in this population of HPV-related head and neck cancer will be evaluated using not only HPV status but also AI-based algorithms—a trend already highlighted by Salvaggio et al. [61]. In their review of studies applying AI and radiomics to CT, MRI, and FDG-PET/CT in oropharyngeal squamous cell carcinoma, the authors focused on HPV prediction and treatment de-intensification, indicating that this approach could facilitate the selection of even more tailored treatment strategies for this demanding patient population in the future.

## 5. Conclusions

In conclusion, our study demonstrates that the prognostic significance of HPV infection varies when outcomes of combined treatment for oral cavity and oropharyngeal cancers are analyzed. On the one hand, we demonstrated better 3-year OS and LRC rates compared to patients with HPV-dependent oropharyngeal cancer. On the other hand, HPV status did not show prognostic significance in the entire study population. At the same time, we demonstrated that HPV-independent patients were given adjuvant treatment after surgery more frequently compared to HPV-dependent patients. Nevertheless, continued research in patients with oropharyngeal and oral cavity cancers remains essential to ensure that, alongside advances in surgical techniques and oncological and radiotherapeutic strategies, the most optimal treatment modalities can be offered in the context of steadily increasing incidence rates.

## Figures and Tables

**Table 1:** Type of adjuvant treatment in patients with squamous cell carcinoma of the oral cavity and oropharynx (N = 51).

N (%)	Type of Adjuvant Treatment
18 (35%)	None
6 (12%)	Radiochemotherapy
24 (47%)	Radiotherapy
2 (4%)	Palliative radiotherapy
1 (2%)	Brachytherapy

**Table 2:** Clinical and morphological characteristics of patients with squamous cell carcinoma of the oral cavity and oropharynx (N = 51).

N (%)	Feature
	Location
28 (55%)	Oral cavity
23 (45%)	Oropharynx
	cT stage
11 (22%)	T1
23 (45%)	T2
16 (31%)	T3
1 (2%)	T4
	cN stage
25 (49%)	N0
2 (4%)	N1
3 (6)	N2a
12 (24%)	N2b
6 (12)	N2c
3 (6)	N3
	Grading
9 (18%)	G1
24 (47%)	G2
18 (35%)	G3
	HPV status
27 (53%)	HPV-dependent
24 (47%)	HPV-independent
	p16
21 (41%)	p16+
30 (59%)	p16−

**Table 3:** Assessment of HPV status with selected clinical and morphological factors in patients with squamous cell carcinomas of the oral cavity and oropharynx (N = 51).

Chi2; *p*	HPV-Independent	HPV-Dependent	Feature
Location			
Chi2 = 0.216; *p* = 0.642	14 (58%)	14 (52%)	Oral cavity
	10 (42%)	13 (48%)	Oropharynx
			Grading
Chi2 = 7.01; *p* = 0.069	1 (4%)	8 (30%)	G1
	15 (62.5%)	9 (33%)	G2
	8 (33.5%)	10 (37%)	G3
cT stage			
Chi2 = 0.96; *p* = 0.810	5 (21%)	6 (22%)	T1
	11 (46%)	12 (44%)	T2
	8 (33%)	8 (30%)	T3
	0	1 (4%)	T4
cN stage			
Chi2 = 2.16; *p* = 0.826	10 (42%)	15 (56%)	N0
	1 (4%)	1 (4%)	N1
	1 (4%)	2 (7%)	N2a
	6 (25%)	6 (22%)	N2b
	4 (17%)	2 (7%)	N2c
	2 (8%)	1 (4%)	N3
Adjuvant treatment			
Chi2 = 4.15; *p* = 0.041	5 (21%)	13 (48%)	No
	19 (79%)	14 (52%)	Yes

**Table 4:** Univariate analysis of factors characterizing the study group and treatment with respect to overall survival (OS).

Factor	Univariate Cox Proportional Hazard Analysis (Wald Statistics)	*p*-Value	Kaplan–Meier Comparison Log-Rank Test	*p*-Value
Gender M vs. F	0.31	0.57	0.54	0.588
Tumor location Oropharynx vs. oral cavity	0.90	0.34	0.96	0.339
T-stage T1-2 vs. T3-4	0.39	0.53	0.65	0.515
N-stage N0-2a vs. N2b+	1.06	0.30	1.06	0.285
HPV status Dependent vs. independent	1.16	0.28	1.09	0.273
Adjuvant treatment No vs. yes	0.47	0.49	0.65	0.516

**Table 5:** Survival analyses with log-rank tests of factors characterizing the study group, treatment and HPV status in oral cavity carcinomas.

Factor	Univariate Cox Proportional Hazard Analysis (Wald Statistics)	*p*-Value	Kaplan–Meier Comparison Log-Rank Test	*p*-Value
Gender M vs. F	0.04	0.85	0.19	0.848
T-stage	0.14	0.71	0.38	0.706
N-stage N0-2a vs. N2b+	0.11	0.74	0.33	0.739
HPV status Dependent vs. independent	0.00	0.96	0.04	0.965
Adjuvant treatment No vs. yes	0.00	0.99	0.00	0.99

**Table 6:** Survival analyses with log-rank tests of factors characterizing the study group, treatment and HPV status in oropharynx carcinomas.

Factor	Univariate Cox Proportional Hazard Analysis (Wald Statistics)	*p*-Value	Kaplan–Meier Comparison Log-Rank Test	*p*-Value
Gender M vs. F	Not estimated *		0.72	0.469
T-stage T1-2 vs. T3-4	Not estimated *		1.37	0.169
N-stage N0-2a vs. N2b+	Not estimated *		1.68	0.092
HPV status Dependent vs. independent	Not estimated *		2.00	0.045
Adjuvant treatment No vs. yes	0.52	0.48	0.64	0.522

*—not estimated because there were no uncensored variables in one subgroup.

## Data Availability

The original contributions presented in this study are included in the article. Further inquiries can be directed to the corresponding author.

## Abbreviations

The following abbreviations are used in this manuscript:

HPV	Human Papilloma Virus
cT stage	clinical Tumor stage
cN stage	clinical Nodal stage
G	Grading
OS	Overall Survival
LRC	Locoregional Control
ECE	Extracapsular invasion
UICC	Union for International Cancer Control
IMRT	Intensity-Modulated Radiation Therapy
ECOG	Eastern Cooperative Oncology Group
CT	Computer Tomography
MRI	Magnetic Resonance Imaging
FDG-PET/CT	Fluorodeoxyglucose-Positron Emission Tomography/Computer Tomography
